# Comparison of Duplex Ultrasound and Digital Subtraction Angiography for Assessing Tibial Vessel Disease

**DOI:** 10.7759/cureus.69327

**Published:** 2024-09-13

**Authors:** Rubén Neris, Ali Kimyaghalam, Kuldeep Singh

**Affiliations:** 1 Vascular Surgery, Trumbull Regional Medical Center, Warren, USA; 2 Vascular Surgery, Staten Island University Hospital, Staten Island, USA

**Keywords:** pvd: peripheral vascular disease, tibial vessel disease, digital substraction angiography, duplex ultrasonography, vascular surgery

## Abstract

Background

Duplex ultrasonography (DUS) is readily available and often used as the first diagnostic test for patients with peripheral artery diseases (PADs). PAD is a disease that affects the general population but more commonly affects diabetics. To date, the role of DUS in the assessment of tibial vessel disease is inconclusive at best. The goal of our study is to assess the validity of DUS in characterizing the presence and severity of tibial diseases via comparison with digital subtraction angiography (DSA) findings.

Methods

This is a single-center retrospective cohort study analyzing three arterial segments (anterior tibial, posterior tibial, and fibular arteries) in patients who received a duplex study followed by DSA within a 30-day period. All arterial segments were graded from normal (Grade 0) to occluded (Grade 4), based on duplex interpretation and directly compared to direct visualization findings from DSA. Using statistical methods, the sensitivity, specificity, positive predictive value (PPV), negative predictive value (NPV), and accuracy of DUS were determined.

Results

A total of 171 tibial vessel segments from 57 enrolled subjects with critical limb ischemia symptoms were analyzed in this study. The agreement between both modalities was poor (Kappa=0.19, p < 0.05), with DUS demonstrating a significant underestimation of vessel pathologies. This is also reflected by the overall sub-optimal sensitivity (23%), specificity (84%), PPV (69%), and NPV (41%) in DUS when compared to DSA results as the gold standard.

Conclusion

Significant disagreements were noted in this study between DUS and DSA findings, primarily significant underestimation of tibial vessel disease by the DUS when compared with the DSA. Caution is advised in the clinical application of DUS in patients with chronic limb-threatening ischemia (CLTI) symptoms and multi-segment tibial vessels due to its demonstrated limitations in this study.

## Introduction

Peripheral artery disease (PAD) occurs in roughly 10% of the American population and is two times more prevalent in the diabetic population [1]. Critical limb-threatening ischemia (CLTI), which is defined as rest pain, non-healing ulceration, or gangrene of the lower extremity, is a more severe manifestation of PAD [2]. In more advanced forms of PAD, such as CLTI, vessel stenosis and/or occlusion are commonly noted in the tibial vessels. Unlike the femoro-popliteal arteries, which are large in caliber (typically 4-8mm in diameter), the tibial vessels are relatively smaller (typically 2-4mm) and commonly calcified, making the vessels more difficult to assess non-invasively [3]. Duplex ultrasonography (DUS) is a valuable tool for the initial diagnosis and subsequent monitoring of PAD. It allows us to see (B-Mode) stenosis and occlusion as well as Doppler analysis of flow velocities and turbulences [4]. A variety of studies have long established duplex ultrasound as a reliable tool for the assessment of femoropopliteal artery stenosis or occlusion [5]. Additionally, duplex ultrasound remains to date the only diagnostic modality that does not require radiation exposure or contrast enhancement. Cost-effectiveness is also important in third-world countries, where it is widely available. As a result, ultrasound plays a critical role in clinical decision-making, especially in the setting of femoropopliteal artery diseases. The role of DUS in the assessment of tibial disease is less established. Currently, the gold-standard assessment technique for tibial vessels remains invasive angiography (i.e., digital subtraction angiography (DSA)) to date [6]. This procedure, however, is associated with significant contrast exposure, potential morbidity secondary to its invasive nature, as well as added costs from advanced equipment requirements. Thus, there exists a profound need for an accurate and reliable non-invasive assessment of the tibial arteries [7]. The goal of our study is to assess the validity of DUS in characterizing the presence and severity of tibial vessel diseases.

## Materials and methods

Study design and subjects

This is a single-center retrospective study. Institutional review board (IRB) approval was granted prior to study initiation within our health system. A total of 57 adult patients in a vascular clinic with symptoms of CLTI, which is classified as the presence of PAD in combination with rest pain, gangrene, or lower limb ulceration lasting for at least two weeks, were enrolled in this study. Exclusion criteria were history of prior intervention and/or history of prior amputation in the lower limb of interest.

Device and interventional procedures

DUS was performed on patients with symptomatic PAD as a screening modality. All examinations were performed by two registered vascular technicians accredited by the American Registry for Diagnostic Sonography (ARDMS). All lower limbs were scanned from the level of groin crease to the level of ankles using a Zonare Z One Pro ultrasound system equipped with a 7-MHz linear probe (Mindray Corp., USA). Peroneal and tibial arteries were scanned with patients in lateral decubitus position. All visualized segments were graded as normal (Grade 0), mild disease with 1% to 19% stenosis (Grade 1), moderate disease with 20% to 49% stenosis (Grade 2), severe disease with 50% to 99% stenosis (Grade 3), or occluded (Grade 4), based on direct visualization of diameter reduction, color flow, spectral characteristics of waveform and velocities on duplex. For ease of categorization, we used a grading system based on peak systolic velocities and velocity ratio.

All enrolled patients underwent conventional DSA within a 30-day period. The decision to use a 30-day period was based on easy follow-up time and avoiding worsening of the condition if waiting longer. Contralateral retrograde common femoral puncture with an up-and-over technique was employed for all angiographic procedures. Nonionic contrast was administered based on standardized vascular laboratory protocol at our institution, with the catheter tip positioned in the superficial femoral artery. A biplane view was obtained in all patients and angiography results were interpreted by a single experienced vascular surgeon subsequently.

Definition

Data was retrospectively collected from the enterprise electronic health record (Sunrise Clinical Manager; Allscripts) between August 2019 and January 2020. Patients’ demographic information and comorbidities were recorded through chart review. Obesity was defined as having a body mass index (BMI) >30. Hypertension, hyperlipidemia, and diabetes status were assessed through clinical chart documentation and medication reconciliation.

Outcome measures

Vessel pathology in lower limbs based on DUS inspection were compared to findings from the following conventional DSA within a 30-day period. Our primary outcome was to compare DUS findings to percent stenosis or occlusion directly visualized on DSA.

Statistics

The data was described using frequencies and percentages for categorical variables and means and standard deviations for quantitative variables. DSA was considered a gold standard diagnostic modality for the purpose of this study. Sensitivity, specificity, positive predictive value (PPV), and negative predictive value (NPV) of DUS were calculated accordingly. Agreement between DUS and DSA was assessed using Cohen’s kappa (ᴋ) statistics. A ᴋ value of 1 indicates perfect agreement, whereas a value of 0 indicates that agreement is no better than chance. All statistical tests were two-sided. P-values less than 0.05 were considered significant. All statistical analyses were performed using IBM SPSS Statistics for Windows, Version 25 (Released 2017; IBM Corp., Armonk, New York, United States).

## Results

Subjects

A total of 57 patients were included in this study. About 23 (40%) subjects were female and 34 (60%) were male. The mean age was 67 years. At the time of intervention, 47 patients (82%) had diabetes, 47 (82%) had hypertension, 24 (43%) were smokers and 21 (38%) had a history of coronary artery disease. All patients suffered from resting claudication (ischemic rest pain) or tissue loss. Table 1 below represents the characteristics of enrolled patients.

**Table 1:** Baseline characteristics of enrolled patients Values presented as no. (%) or mean (± standard deviation) where appropriate. Age refers to the age at study enrollment.

Variables	N = 57
Mean age	67 (±12)
Male	34 (60%)
Diabetes mellitus	47 (82%)
Hypertension	47 (82%)
Current smoker	24 (43%)
Coronary artery disease	21 (38%)
Critical limb ischemia	57 (100%)

Findings

A total of 171 tibial vessels were analyzed in this study. With DUS, a total of 98 arterial segments were identified as normal, 10 were identified as mildly diseased (Grade 1), 20 were identified as moderately diseased (Grade 2), 19 were identified as severely diseased (Grade 3) and 13 were identified as completely occluded (Grade 4). In comparison, DSA identified nine normal segments (Grade 0), 17 mildly diseased segments (Grade 1), 46 moderately diseased segments (Grade 2), 61 severely diseased segments (Grade 3), and 38 occluded segments (Grade 4). Table 2 below is a graphic representation of the segmental stenosis between the DUS and DSA.

**Table 2:** Segmental stenosis measured by DUS versus DSA Due to technical difficulty, two anterior tibial segments, three posterior tibial segments, and six peroneal segments were not visualized at the time of the DUS exam. DUS: doppler ultrasonography; DSA: digital subtraction angiography; AT: anterior tibial artery; PT: posterior tibial artery

N = 57	DUS AT	DSA AT	DUS PT	DSA PT	DUS peroneal	DSA peroneal
Normal (Grade 0)	33	2	30	3	35	4
Mild (Grade 1)	2	7	6	7	2	3
Moderate (Grade 2)	11	15	5	18	4	13
Severe (Grade 3)	4	14	9	17	6	30
Occlusion (Grade 4)	5	19	4	12	4	7

The sensitivity, specificity, PPV, NPV, and agreement of DUS, as compared to DSA, are presented in Table 3.

**Table 3:** Sensitivities, specificities, PPV, NPV, and kappa statistics for DUS in detecting ≥50% stenosis or occlusions in tibial vessels 95% confidence interval: 70 ± 1.3. The number of correlated pairs is 20. PPV: positive predictive value; NPV: negative predictive value; DUS: doppler ultrasonography; AT: anterior tibial artery; PT: posterior tibial artery

	Sensitivity	Specificity	PPV	NPV	Agreement
AT	29%	100%	100%	52%	ᴋ = 0.36
PT	24%	76%	64%	46%	ᴋ = 0.22
Peroneal	16%	71%	60%	24%	ᴋ = 0.13
Overall	23%	84%	69%	41%	ᴋ = 0.19

To demonstrate how the degree of stenosis is interpreted in the lower extremities using the DUS, we decided to use a widely used method of interpretation from the *Seminars in Vascular Surgery*, as shown in Table 4.

**Table 4:** Degree of stenosis Reference: [4]

Degree of stenosis	Peak systolic velocity (cm/s)	Velocity ratio
<20%	<150	<1.5
20-49%	150-200	1.5-2.0
50-80%	200-300	2.0-4.0
>80%	>300	>4.0
Occlusion	No flow detected in lumen	N/A

**Figure 1:**
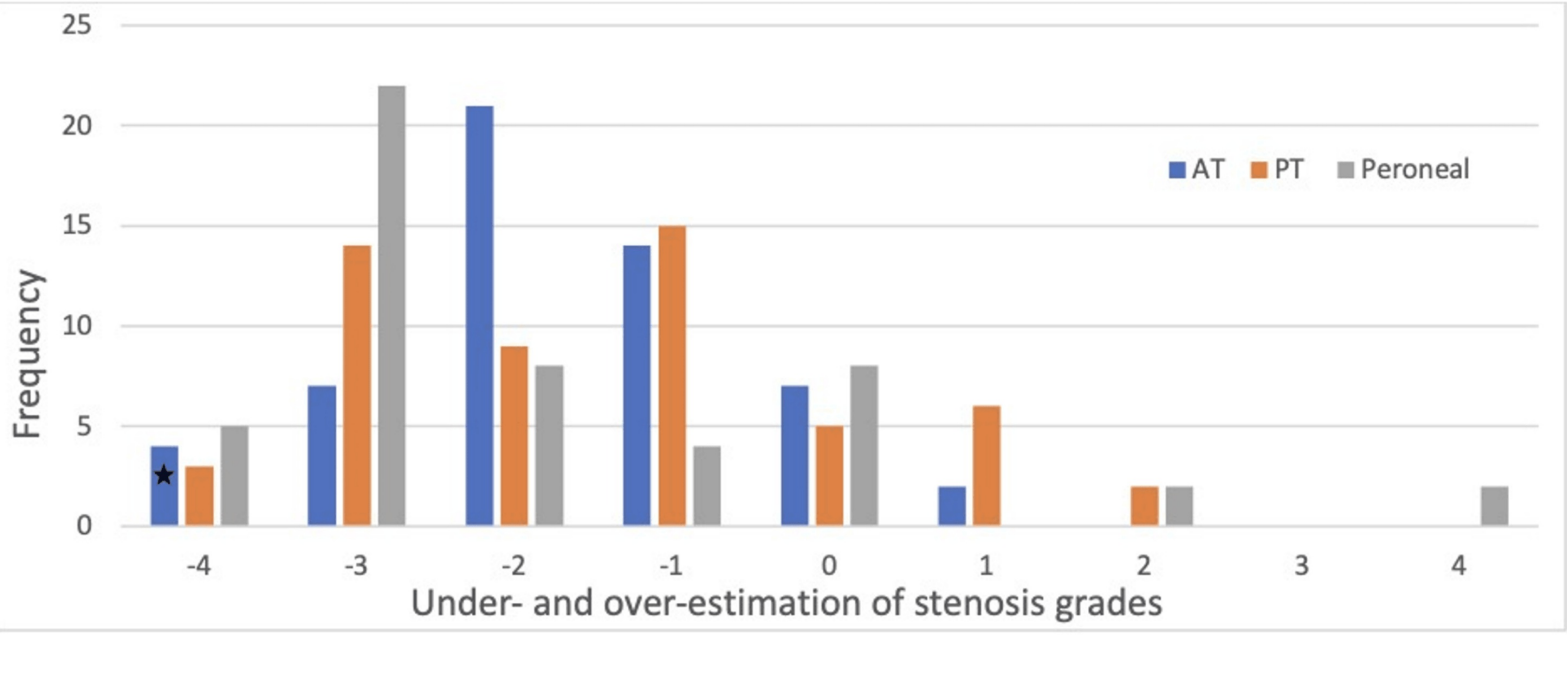
Under-estimation and over-estimation of segmental stenosis by DUS 0, findings agreed between DUS and DSA; -4, -3, -2, -1, numbers of grades of underestimation by DUS; +1, +2, +3, +4, number of grades of over estimation by DUS. For instance, if an anterior tibial segment (blue coded) is interpreted as grade 0 stenosis by DUS and confirmed as grade 4 stenosis by DSA, the degree of underestimation by DUS in this case will be -4 and will be counted in the frequency column marked above (star). DUS: doppler ultrasonography; AT: anterior tibial artery; PT: posterior tibial artery

A closer look at our data reveals that DUS underestimated the degree of stenosis by four grades in 12 segments, by three grades in 43 segments, by two grades in 38 segments, and by one grade in 33 segments. DUS and DSA results completely agreed on the grading of stenosis in 20 arterial segments. Figure 1 illustrates the degree of under-and over-estimation by DUS characterizing stenosis of individual tibial vessels.

## Discussion

In this study retrospectively comparing DUS and DSA for the diagnosis of tibial vessel diseases, we demonstrated significant discrepancies between the two diagnostic modalities. It comes as no surprise that this discrepancy exists, as the deep anatomic positions and small vessel calibers may compromise insonation in duplex scanning even in the most experienced ultrasonographers’ hands [8]. Moreover, the comorbidities and anatomic features specific to each individual patient, such as obesity, arterial calcification, skin ulceration, and edema may further limit the utility of ultrasound in assessing the tibial vessels.

While the validity of ultrasound in the femoropopliteal arteries has been well-established via a variety of comparisons between DSA and DUS, fewer such studies of tibial vessels have been reported recently in the literature. Like our findings, previous studies demonstrated relatively low k values (0.25 by Favaretto et al. and 0.59 by Katsamouris et al.) indicating poor agreement in diagnosing tibial diseases by either modality [9,10]. In this same vascular territory, DUS has insufficient sensitivity (24% by Favaretto et al.) and specificity (40% by Mustapha et al.) when compared to the gold standard of DSA [6,9]. However, imaging is not limited to just DUS and DSA. Other modalities like magnetic resonance (MR) and computer tomography (CT) can be used to visualize the calf arteries.

Conclusions derived from these studies illustrated that duplex scanning and angiography should be considered complementary techniques in providing functional as well as anatomical information on the arterial distribution studied. Decisions for therapy should not be solely based on DUS findings [11]. In this setting, the combination of DUS and DSA results provides useful information that optimizes the overall decision-making for subsequent intervention [12,13]. In addition, further studies can focus on other modalities including ankle brachial index/pulse volume recordings (ABI/PVR) and toe pressure.

Our results further demonstrated that, in our patient population where severe CLTI symptoms and multi-level diseases are common, DUS tends to significantly underestimate degrees of stenosis or occlusion status in tibial vessel segments. While vessel caliber and calcium burden may be partially responsible, the concomitant presence of proximal or adjacent diseases also limit the accuracy of DUS and renders underestimation of disease severity from its interpretation - a phenomenon which has been described elsewhere in the literature.

Allard et al., in a study published in 1994, found that a significant number of duplex mis-classifications underestimating the degrees of stenosis were associated with lower extremity tandem lesions [14]. In another study lead by Brown et al., turbulent flows within an in vivo model, such as those generated from tandem lesions in arterial segments, were thought responsible for as much as a 50% underestimation of DUS assessed stenosis severity in controlled laboratory conditions [15]. Due to this interference introduced by tandem lesions, currently established protocols may be unreliable when used to assess the severity of distal stenotic lesions. Specifically, the peak systolic velocity ratio of 2 as a threshold for diagnosing 50% stenosis used in the present study appears arbitrary and were based on theoretical calculations from ideal settings [16]. Some authors, such as Macharzina et al., have advocated for increasing the peak systolic velocity ratio threshold to 2.6 when establishing a stenosis of >50% to account for the effect of lesions in tandem [17]. However, it is important to keep in mind there are major differences between the present study and the cited literatures, i.e., our study focused on tibial vessels whereas the quoted data reflected results from either larger-sized supra-popliteal vasculature or controlled laboratory models.

This study is subject to several limitations. The retrospective, non-randomized nature of this study subjects itself to the inherent weakness of such analyses. For example, the angiographic technique utilized was not standardized. As a result, the location of the distal catheter tip, the injection method, and contrast amount may have differed significantly amongst the subjects. Similarly, sonographer skill differences, variation in ultrasound techniques, and the extent to which the tibial vessels were interrogated may have all impacted the results. Furthermore, the sample size remains small, and the definition of arterial segments remains arbitrary in our study. Our results did not consider common anatomical variations among studied subjects and the impact such anomalies have on DUS and DSA findings.

## Conclusions

There exist significant differences between DUS and DSA in diagnosing tibial vessels PAD. Caution is advised, therefore, in the clinical application of DUS in patients with CLTI symptoms and multi-segment tibial vessels due to its demonstrated limitations in this study. Given the findings shown in this study, we recommend using the DUS as a screening method initially. If further workup needs to be done, the DUS must be complemented with a more accurate diagnostic test, such as DSA. Using the DUS as the only test for the diagnosis of tibial vessels with PAD in this population will result in an undesirable underestimation of the patient's condition and, therefore, all the consequences that inappropriate diagnosis/treatment can bring. Finally, future studies should aim to examine DUS’s ability to safely and accurately guide therapeutic vascular interventions in this patient population with tibial vessels.
